# Targeting Serotonin With Common Antidepressants Induces Rapid Recovery From Cytopenia

**DOI:** 10.1093/stcltm/szac055

**Published:** 2022-08-10

**Authors:** Guillemette Fouquet, Julien Rossignol, Nicolas Garcelon, Olivier Hermine, Francine Côté, Tereza Coman

**Affiliations:** Institut Imagine, INSERM U1163, Université Paris Cité, Paris, France; Hématologie, Hôpital Necker, APHP, Paris, France; Hématologie, Centre Hospitalier Sud Francilien, Corbeil-Essonnes, France; INSERM U1016, Institut Cochin, Institut National de la Santé et de la Recherche Médicale, Centre National de la Recherche Scientifique, Paris, France; Institut Imagine, INSERM U1163, Université Paris Cité, Paris, France; Hématologie, Hôpital Necker, APHP, Paris, France; Institut Imagine, INSERM U1163, Université Paris Cité, Paris, France; Institut Imagine, INSERM U1163, Université Paris Cité, Paris, France; Hématologie, Hôpital Necker, APHP, Paris, France; Institut Imagine, INSERM U1163, Université Paris Cité, Paris, France; INSERM U1016, Institut Cochin, Institut National de la Santé et de la Recherche Médicale, Centre National de la Recherche Scientifique, Paris, France; Institut Imagine, INSERM U1163, Université Paris Cité, Paris, France; INSERM U1016, Institut Cochin, Institut National de la Santé et de la Recherche Médicale, Centre National de la Recherche Scientifique, Paris, France; Hématologie, Institut Gustave Roussy, Villejuif, France

**Keywords:** serotonin, antidepressants, cytopenia, autologous hematopoietic stem cell transplantation, chemotherapy, G-CSF

## Abstract

The hematopoietic system uses several, yet undiscovered, factors to adapt to stresses such as chemotherapy, infections, or bleeding. Serotonin is commonly known as a neurotransmitter but is also produced and used in peripheral organs. In particular, we have shown that serotonin synthesized in the bone marrow is necessary for erythroid progenitors’ survival and proliferation. Serotonin levels can be increased by FDA approved antidepressants called selective serotonin reuptake inhibitors (SSRI). In this work, we report a previously unknown role of SSRI in the recovery of cytopenia, after autologous hematopoietic stem cell transplantation in patients and after sub-lethal irradiation in mice. We also observed an unexpected cooperation between SSRI and G-CSF on the improvement of the 3 hematopoietic lineages. Of note, SSRI do not seem to affect blood cells production in the absence of stress-induced hematopoiesis. We propose that the serotonergic system could be a valuable therapeutic target in stress-induced cytopenia, especially as a rescue after radiation or chemotherapy.

## Graphical Abstract

**Figure:**
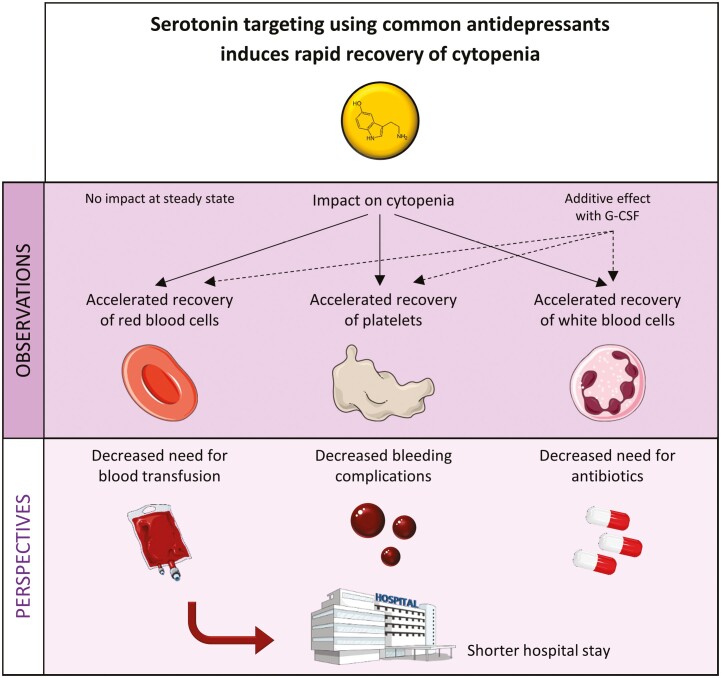
Targeting the serotonergic system as a novel strategy for reducing chemotherapy-induced cytopenia and their complications in human.

**Lessons Learned**

• Serotonin is a new factor in erythropoiesis, with pro-survival and proliferative functions.
• Selective serotonin reuptake inhibitors (SSRIs), common antidepressants, enhance recovery from cytopenia after irradiation or chemotherapy.
• The serotonergic system may be a valuable therapeutic target for reducing radiation- or chemotherapy-induced cytopenia in human.

**Significance Statement**

We recently identified serotonin as a new factor in erythropoiesis, with pro-survival and proliferative functions on mouse and human erythroid progenitors. Here, we describe that selective serotonin reuptake inhibitors (SSRIs), common antidepressants, enhance recovery from cytopenia after irradiation in mice and autologous stem cell transplantation in humans. We believe that the serotonergic system may be a valuable therapeutic target for reducing radiation- or chemotherapy-induced cytopenia, through the use of SSRI.

## Introduction

Hematopoiesis is a highly regulated system where multiple, insufficiently understood factors orchestrate the self-renewal of bone marrow stem cells and their differentiation into blood cells. Following acute stresses such as infections, inflammation, chemotherapy, or radiation, the hematopoietic system quickly adapts through “emergency” or “stress” hematopoiesis.^1-3^ Identifying factors that enhance hematopoiesis and limit the extent and duration of cytopenia is extremely important, because treatment-induced cytopenia increases morbidity, mortality, and economic impacts.

The promoting effect of serotonin in hematopoiesis has been suggested before, as in an early work on human hematopoietic stem cells,^4^—among other very interesting studies reviewed recently by our group.^5^

More recently, we identified, in human and murine erythroid progenitors, a functional cell-autonomous serotonergic network with pro-survival and proliferative functions.^6^ Administration of selective serotonin reuptake inhibitors (SSRIs) such as fluoxetine, a common antidepressant, can rescue the anemic phenotype in mice models.^6^

Here we hypothesize that serotonin also has a role on other hematopoietic lineages and that the serotonergic system may be a valuable therapeutic target for reducing radiation- or chemotherapy-induced cytopenia.

## Methods

### Mice Models

Mice were housed 5 per cage with food and water ad lib and a 12-hour:12-hour light:dark cycle. C57BL/6 WT mice (males and females 8-10 weeks old, randomly assigned to experimental groups) were purchased from Janvier Labs. Animal experiments were performed per recommendations of the competent French ethics committee.

For aplasia experiments, we submitted C57BL/6 WT mice to sublethal irradiation (5.5 Gy on day 0 [D0]). Peripheral blood was collected in EDTA tubes and analyzed using a ProCyte Dx Hematology Analyzer (IDEXX, Westbrook, ME, USA). We compared established murine biological parameters in mice treated with 20 mg/kg/day fluoxetine (Prozac ) administered in drinking water, starting D −1 (ie, day before irradiation) unless stated otherwise, to those in control mice—with or without granulocyte colony-stimulating factor (G-CSF, filgrastim).^7, 8^ G-CSF was administered intraperitoneally at 5 µg/kg/day from D5 to the end of the experiment.

### Human Cohorts

A database^9^ was used to select patients who underwent an autologous hematopoietic stem cell transplant (ASCT) in the adult hematology department of Necker Hospital (Paris, France) between 2008 and 2018. We compared 22 patients under SSRIs during their ASCT to 66 controls, matched according to number of injected CD34^+^ cells/kg, age, sex, conditioning chemotherapy, pathology, extent of response before transplantation, previous lines of chemotherapy, and year of transplant.

In a second cohort, we compared 30 patients treated with SSRIs at the time of CD34^+^ mobilization to 176 controls, matched according to the same factors (except injected CD34^+^ cells/kg and conditioning chemotherapy) in addition to means of mobilization, ie, G-CSF, plerixafor, or high-dose cyclophosphamide. Eighteen patients given SSRIs and 57 controls belonged to both cohorts. The study complied with the Declaration of Helsinki.

### Flow Cytometry

Flow cytometry antibodies were purchased from Sony and BD Biosciences. Cells were analyzed on a FACSCanto II (BD Biosciences, San Jose, CA, USA) or an SP6800 Spectral Analyzer (Sony Biotechnology, San Jose, CA, USA) using FlowJo v. X.0.7 (Tree Star, Ashland, OR, USA). Supplementary Fig. 1 shows gating strategies.

### Statistical Analysis

Statistical analysis was conducted with GraphPad Prism (GraphPad Software, San Diego, CA, USA). As appropriate, data are presented in figures as mean and SEM, and unpaired *t*-tests or Mann-Whitney tests were performed. An asterisk indicates significance (*P* < .05).

## Results and Discussion

### SSRI Treatment Hastens Recovery from Chemotherapy-Induced Cytopenia Associated with ASCT in Humans

**Figure 1.**
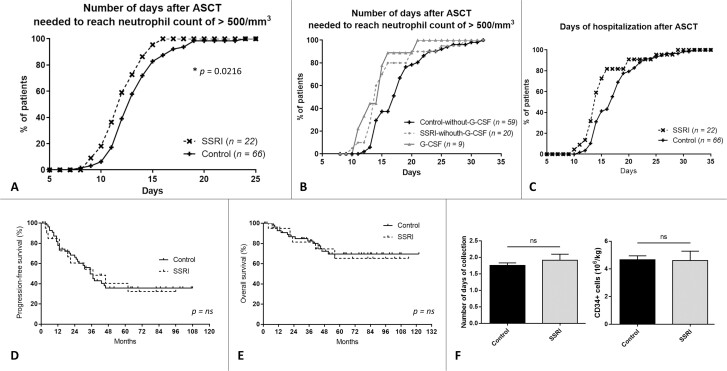
SSRIs hasten recovery from ASCT-related cytopenia in humans. (**A**) Recovery from neutropenia after ASCT in retrospective cohort of 22 patients treated with SSRIs and 66 paired controls. Mean number of days elapsed before reaching neutrophil count of > 500/mm^3^ was 12.2 in SSRI group and 14.3 in control group (*P* = .0216). (**B**) Recovery from neutropenia after ASCT in same cohort, according to treatment received: G-CSF, SSRI without G-CSF, or none (control without G-CSF). (**C**) Days of hospitalization, (**D**) progression-free survival, and (**E**) overall survival after ASCT in same cohort, for SSRI and control groups. (**F**) Number of days CD34 + cells were collected (left graph) and number of CD34 + cells collected (right graph), prior to ASCT, in second retrospective cohort, for SSRI and control groups. Abbreviations: ASCT, autologous stem cell transplant; G-CSF, granulocyte colony-stimulating factor; ns, not significant.

ASCT is a standardized procedure inducing profound cytopenia that is closely monitored and of predictable duration, which facilitates recruitment of a homogenous cohort of patients. We included 22 patients taking SSRIs (paroxetine: 6; escitalopram: 5; citalopram: 3; venlafaxine: 3; fluoxetine: 3; sertraline: 2) over a median period of 27 days (range: 1-2514) before ASCT. All were matched for factors known to modulate recovery from aplasia. The mean number of neutropenic days (neutrophil count: ≤500/mm^3^) was 12.2 in the SSRI group and 14.26 in the control group (*P* = .0216) (Fig. 1A). Groups did not differ significantly in terms of red cell or platelet transfusions (data not shown).

As our previous work^6^ suggested interaction between serotonin and erythropoietin, we wondered whether serotonin could augment effects of other known hematopoietic growth factors. Post-ASCT G-CSF administration is a common, albeit center-dependent, treatment demonstrably shortening the duration of aplasia and hospitalization. Two (9.1%) SSRI group and 7 (10.6%) control group patients received G-CSF. We compared this G-CSF group (*n* = 9) to the remaining SSRI (*n* = 20) and control (*n* = 59) patients. As expected, G-CSF quickened recovery from aplasia, but remarkably, SSRI treatment alone was almost as effective as G-CSF (Fig. 1B). At D15, 78% of the G-CSF subset and 70% of the SSRI-without-G-CSF subset had neutrophil counts of > 500/mm^3^, vs 37% of the control-without-G-CSF subset. Of note, among the 9 patients in the G-CSF group, 2 patients also received SSRI and 9 patients did not. We did not observe any significant difference in the duration of aplasia between G-CSF alone, G-CSF + SSRI or SSRI alone; however, the limited number of patients does not allow to draw any definitive conclusion.

The mean number of days of post-ASCT hospitalization was 15.5 in the SSRI group and 17.6 among controls (*P* = .0545) (Fig. 1C). At D15, 73% of SSRI group patients were discharged from the hospital vs 41% of control patients. SSRI therapy was not associated with a higher rate or quicker relapses of underlying malignant hemopathies: median progression-free survival was 37 months in the SSRI group vs 35 months in the control group (not significant) (Fig. 1D), and median overall survival was not reached (Fig. 1E).

Given the suggested role of serotonin in stem cell mobilization,^10^ we wished to investigate how SSRIs affect mobilization of CD34^+^ cells. In a second matched SSRI-vs-control cohort, we did not find significant differences in numbers of CD34^+^ cells collected, graft viability at freezing or thawing, yield, numbers of days of mobilization, or achievement of CD34^+^ objectives (Fig. 1F). Patients who received SSRI-mobilized stem cells did not recover faster than controls.

However, our human data are based on a retrospective analysis, and must be interpreted with caution as they could be the subject of various biases. Our findings should be confirmed in prospective studies.

### In Mice, SSRI Treatment (Fluoxetine), With or Without G-CSF, Speeds Recovery from Irradiation-Induced Cytopenia

**Figure 2.**
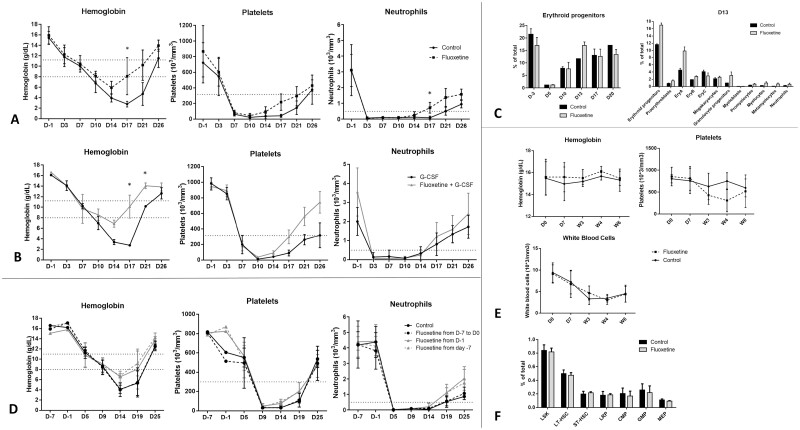
SSRI (fluoxetine), alone or with G-CSF, hastens recovery from irradiation-induced cytopenia in mice. (**A**), (**B**), (**D**): Horizontal lines represent lower limit of normal range for hemoglobin (11 g/dL), platelets (300 × 10^3^/mm^3^), and neutrophils (0.5 × 10^3^/mm^3^), and threshold for profound anemia (8 g/dL), in mice.^7, 8^ (A) In vivo rescue experiments with fluoxetine: hemoglobin (g/dL), platelet (10^3^/mm^3^), and neutrophil (10^3^/mm^3^) levels in WT mice (*n *= 7) treated with fluoxetine or placebo (control) from D − 1 (ie, 1 day before sublethal irradiation) until end of experiment; data from 3 independent experiments. (**B**) In vivo rescue experiments with G-CSF, alone or with fluoxetine: hemoglobin (g/dL), platelet (10^3^/mm^3^), and neutrophil (10^3^/mm^3^) levels in WT mice (*n *= 5) treated with G-CSF, alone or with fluoxetine, from D − 1 before sublethal irradiation until end of experiment; data from 3 independent experiments. (**C**) Flow cytometry analysis of hematopoietic progenitor cells in bone marrow of WT mice (*n* = 5) treated with fluoxetine or placebo (control) from D − 1 before sublethal irradiation. Change over time for erythroid progenitors shown on left; D13 data for all progenitors shown on right. *Erythroid progenitors: cKIT*^*+*^*, Ter119*^*+/int*^*, CD71*^*+/−*^*; proerythroblasts: cKIT*^*+*^*, CD71*^*+*^*, Ter119*^*int*^*; EryA: cKIT*^*+*^*, Ter119*^*+/int*^*, CD71*^*+*^*, FSC-A*^*high*^*; EryB: cKIT*^*+*^*, Ter119*^*+/int*^*, CD71*^*+/int*^*, FSC-A*^*low*^*.; EryC: cKIT*^*+*^*, Ter119*^*+/int*^*, CD71*^*−*^*, FSC-A*^*low*^*.; megakaryocytes: Ter119*^*−*^*, cKIT*^*+,*^*CD41*^*+*^*; granulocyte progenitors: lineage-negative, removal of cKIT*^* + *^*CD34*^* − *^*cells, maturation from myeloblasts to neutrophils according to cKIT and Ly6G markers* (see Supplimentary Fig. 1A). (D) Timing of fluoxetine treatment in rescue experiments in mice: hemoglobin (g/dL), platelet (10^3^/mm^3^), and neutrophil (10^3^/mm^3^) levels in WT mice (*n* = 5) treated with placebo (control) or fluoxetine from D − 7 to D0 (ie, day of irradiation), from D − 7 on, or from D − 1 on; data from 3 independent experiments. (E) Hemoglobin, platelet, and neutrophil levels in WT mice (*n* = 7) treated with placebo (control) or fluoxetine for 0 days, 7 days, 3 weeks, 4 weeks, or 6 weeks, at steady state without any other intervention. (**F**) Flow cytometry analysis of hematopoietic stem and progenitor cells in bone marrow of WT mice (*n* = 7) treated with placebo (control) or fluoxetine for 4 weeks, at steady state without any other intervention. Abbreviations: LSK, Lin^−^, Sca1^+^, CD117^ + ^cells; LT-HSC and ST-HSC, long-term (LSK, CD150^+^, CD48^−^) and short-term (LSK, CD150^+^, CD48^+^) hematopoietic stem cells; LRP, lineage-restricted progenitors (LSK, CD150^−^, CD48^+^); CMP, common myeloid progenitors (LK, CD34^+^, CD16/32^int^); GMP, granulocyte macrophage progenitors (Lin^−^, Sca1^−^, CD117^+^, CD34^+^, CD16/32^high^); MEP, megakaryocyte erythroid progenitors (Lin^−^, Sca1^−^, CD117^+^, CD34^−^, CD16/32^low^) (see Supplementary Fig. 1B); G-CSF, granulocyte colony-stimulating factor; W, week; WT, wild type.

WT mice recovered faster from neutropenia, anemia, and thrombopenia after sublethal irradiation, when administered fluoxetine starting the day before (Fig. 2A). As in our patient cohort, we studied the combination of fluoxetine and G-CSF in vivo. In sublethally irradiated mice, we observed an additive effect between fluoxetine administered from D −1 and G-CSF on the recovery of the 3 myeloid lineages. At D17 post-irradiation, mean values for mice given fluoxetine and G-CSF and for control mice were, respectively, as follows: hemoglobin, 10.5 and 4.0 g/dL (*P* < .0001); neutrophils, 1.220 and 0.253.10^3^/mm^3^ (*P* = .0003); and platelets, 308 and 84.10^3^/mm^3^ (*P* = .0009) (Fig. 2B, Supplementary Table S1). In addition, fluoxetine slightly increased committed erythroid and granulocyte progenitors in the bone marrow—but not stem cells—, around 13 days after irradiation (Fig. 2C right). We did not observe any clear difference in megakaryocytes number and differentiation. At other time points, the proportion of progenitors of the 3 lineages were similar between mice treated with fluoxetine or placebo, as shown for erythroid progenitors in Fig. 2C left. We also sought to determine whether fluoxetine’s effects were due to conferral of protection from irradiation, but no benefit from early fluoxetine administration during the 7 days preceding irradiation was observed (Fig. 2D).

Finally, fluoxetine administered to mice under steady-state conditions did not alter blood counts during a 6-week follow-up period (Fig. 2E). It did not affect bone marrow hematopoietic stem and progenitor cells at steady-state over time (Fig. 2F). In humans, long-term SSRI treatment for depression reportedly has little to no effect on steady-state hematopoiesis.^11^ These data suggest that, in the absence of hematopoietic stress, fluoxetine might not appreciably alter hematopoiesis.

## Conclusion

We describe SSRI-enhanced recovery from cytopenia after irradiation (mice) and ASCT (humans), and strengthened recovery of myeloid lineages through combination of an SSRI and G-CSF. Thus, the serotonergic system may be a valuable therapeutic target in therapy-induced aplasia, warranting investigation of the treatment of such hematopoietic deficiencies with common antidepressants.

## Supplementary Material

szac055_suppl_Supplementary_Figure

szac055_suppl_Supplementary_Figure_Legend

szac055_suppl_Supplementary_Table_1

## Data Availability

The data underlying this article will be shared on reasonable request to the corresponding author
