# Supplementary material for: Targeting Serotonin With Common Antidepressants Induces Rapid Recovery From Cytopenia
Source: Stem Cells Transl Med. 2022 Aug 10;11(9):927–31. doi: 10.1093/stcltm/szac055 (PMC9492259; doi:10.1093/stcltm/szac055)
Supplement: szac055_suppl_Supplementary_Figure [file szac055_suppl_supplementary_figure.pdf]

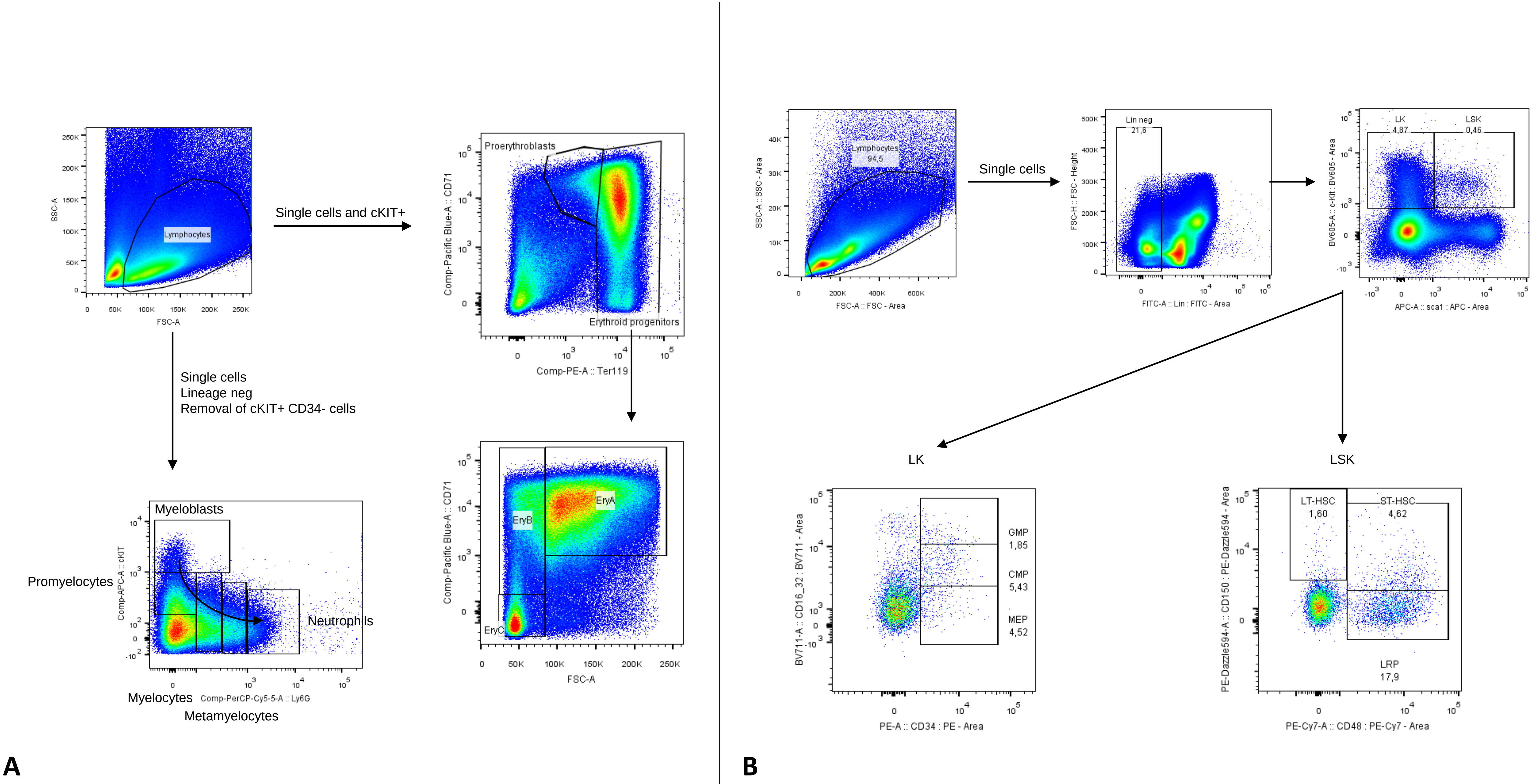

Supplementary Figure 1. Flow cytometry gating strategies.

[illegible]

Supplemental Table 1. Mean values 17 days after irradiation in pooled mice experiments

Treatment	Hemoglobin, g/dL	Platelets, $10^3/mm^3$	Neutrophils, $10^3/mm^3$
Control	4	84	0.253
Fluoxetine	7.3	140	0.602
G-CSF	8	263	0.813
G-CSF + fluoxetine	10.5	308	1.220

G-CSF, granulocyte colony-stimulating factor.
