# Supplementary material for: Targeting Serotonin With Common Antidepressants Induces Rapid Recovery From Cytopenia
Source: Stem Cells Transl Med. 2022 Aug 10;11(9):927–31. doi: 10.1093/stcltm/szac055 (PMC9492259; doi:10.1093/stcltm/szac055)
Supplement: szac055_suppl_Supplementary_Figure_Legend [file szac055_suppl_supplementary_figure_legend.docx]

**Supplemental Figure 1. Flow cytometry gating strategies.** For analyses of murine bone marrow described in (A) Figure 2C and (B) Figure 2F.
