# Supplementary material for: Targeting Serotonin With Common Antidepressants Induces Rapid Recovery From Cytopenia
Source: Stem Cells Transl Med. 2022 Aug 10;11(9):927–31. doi: 10.1093/stcltm/szac055 (PMC9492259; doi:10.1093/stcltm/szac055)
Supplement: szac055_suppl_Supplementary_Table_1 [file szac055_suppl_supplementary_table_1.docx]

Supplemental Table 1. Mean values 17 days after irradiation in pooled mice experiments

| Treatment | Hemoglobin, g/dL | Platelets, 10^3^/mm^3^ | Neutrophils, 10^3^/mm^3^ |
| --- | --- | --- | --- |
| Control | 4 | 84 | 0.253 |
| Fluoxetine | 7.3 | 140 | 0.602 |
| G-CSF | 8 | 263 | 0.813 |
| G-CSF + fluoxetine | 10.5 | 308 | 1.220 |

*G-CSF, granulocyte colony-stimulating factor.*
